# A randomized comparison between ultrasound-guided costoclavicular and infraclavicular block for upper extremity surgery

**DOI:** 10.3906/sag-2011-126

**Published:** 2021-08-30

**Authors:** Sevim CESUR, Ahmet Murat YAYIK, Ayşe Nur DAŞ, Ali AHISKALIOGLU

**Affiliations:** 1 Department of Anesthesiology and Reanimation, Kocaeli University, School of Medicine, Kocaeli Turkey; 2 Department of Anesthesiology and Reanimation, Atatürk University, School of Medicine, Erzurum Turkey; 3 Department of Anesthesiology and Reanimation, Regional and Training Research Hospital, Erzurum Turkey

**Keywords:** Costoclavicular approach, brachial plexus, infraclavicular block, sensorimotor blockade, upper extremity surgery

## Abstract

**Background/aim:**

This study compared ultrasound guided costoclavicular (CC) and lateral sagittal infraclavicular (LS) brachial plexus block in patients undergoing upper extremity surgery.

**Materials and methods:**

A total of 80 patients undergoing upper extremity surgery were randomly classified into two groups: Group CC (costoclavicular (n = 40)) and Group LS (lateral sagittal infraclavicular (n = 40)). Both groups received a 25 mL containing a mixture of 1% lidocaine and 0.25% bupivacaine. A blinded observer recorded the block onset time and decided which patients who were admitted to the operation room needed general anesthesia or rescue block or without any iv. narcotics for the surgical procedure.

**Results:**

The sensorimotor onset time was found to be faster in the CC group [(15.95 2.97) min] compared to the LS group [(17.72 4.15)min]. There was a statistically significant difference between two groups in terms of sensorimotor onset time (p = 0.031). There was no difference between two groups in terms of the block performance times and post-block motor block dissolution times.

**Conclusion:**

The CC approach provides faster onset of sensorimotor blockade than LS approach when the 4 major terminal nerves of the brachial plexus were evaluated.

## 1. Introduction

Ultrasound-guided infraclavicular block is a regional technique frequently used by anesthesiologists in upper extremity surgery by blocking the brachial plexus at the level of cords at the second part of the axillary artery. Infraclavicular block can be applied with different approaches. Lateral sagittal infraclavicular block (LS) has been defined as effective and safe; however, some limitations of the procedure have also been reported such as deep location of the brachial plexus through LS approach, variational location of cords around the axillary artery; thus, it might be impossible to see all three cords by single sagittal ultrasound window [1–6]. In 2015, Karmakar et al. [3] have recently described a new costoclavicular block (CC) by which the brachial plexus is targeted immediately caudal to the clavicle in the costoclavicular space. Compared to LS, CC has some advantages such as the cords being superficial and clustered together [3].

In this study, our primary outcome was to compare the CC approach and the LS approach in the terms of onset time the sensorimotor blockade. Our secondary outcomes were to compare performance time according to LS, which we frequently performed, and to evaluate the activity, effectivity and the incidence of adverse effects of both blocks. 

## 2. Materials and methods

After obtaining an approval from the local ethics committee (Erzurum BEAH KAEK 2018/01-03), 80 patients aged 18 years and above and with ASA I-III and BMI 18-40 kg/m^ 2 ^and who had undergone an emergency or elective hand, wrist, forearm and elbow surgery were included in the study. The procedure was explained to each patient, and a written informed consent was obtained. Patients who did not prefer the block procedure or who had skin infection at the location of the puncture, coagulopathy, sepsis, local anaesthetic (LA) allergy and musculocutaneous, radial, ulnar or median neuropathy were excluded from the study.

Patients were randomly allocated to two groups on a randomization computer programme: Group- CC (costoclavicular) and Group- LS (infraclavicular). Standardized monitoring was applied to all patients who were admitted to the regional anesthesia room, and an intravenous (iv) access was placed on the contralateral upper extremity, and patients were sedated with 0.03 mg.kg^–1^ iv midazolam. All these blocks were performed by the same anaesthesiologist (SC).

### 2.1. Ultrasound-guided lateral sagittal approach 

**Figure 1:**
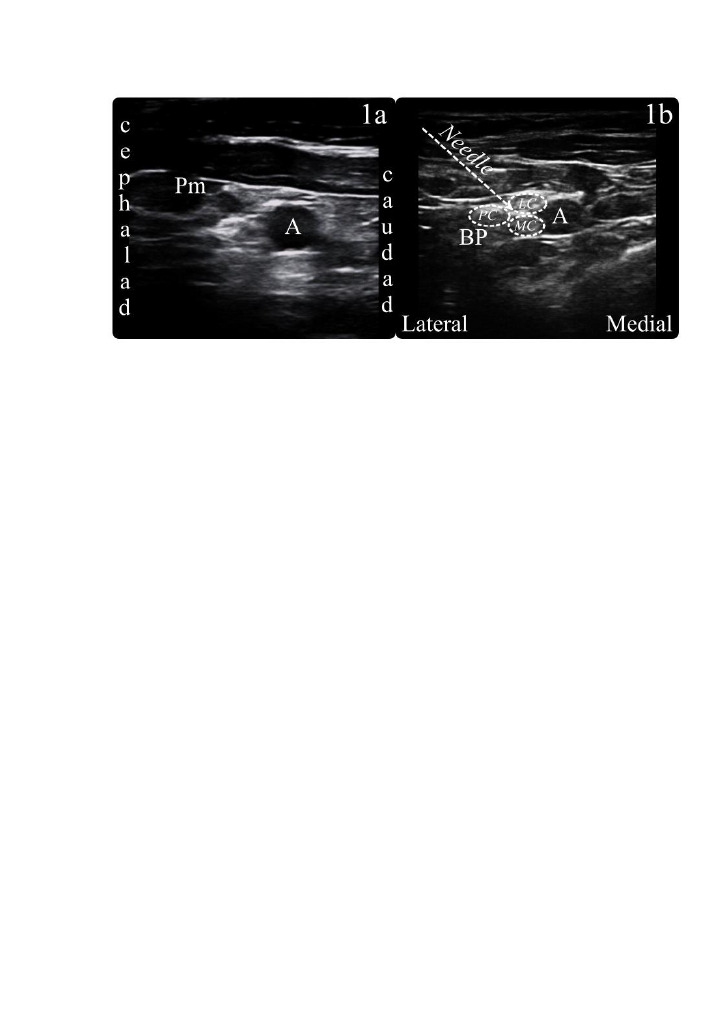
Sonographic view of both blocks. 1a. Sonographic view of the brachial plexus in the lateral infraclavicular fossa; 1b. Sonographic view of the brachial plexus in the costoclavicular space. Pm: pectoralis minör muscle; A: axillary artery; BP: brachial plexus LC: Lateral cord; MC: Medial cord; PC: Posterior cord.

The skin through which the block would be performed was wiped using an antiseptic solution while the patient was in the supine position. The linear ultrasound probe was prepared in a sterile way. In the LS group, blocks were performed ultrasound-guided using a single needle entry. Sagittal ultrasound image revealed the second part of the axillary artery, and the ultrasound image was optimized (Figure 1a). Using the in-plane technique the needle was entered to a site between the posterior cord and the axillary artery. After negative aspiration, 1–2 mL of saline 0.9% was injected to confirm the needle position. A LA of 25 mL containing a mixture of 1% lidocaine and 0.25% bupivacaine was administered around the axillary artery in a ‘U’ shape by wrapping the axillary artery.

### 2.2. Ultrasound-guided costoclavicular approach 

The extremity to be blocked was placed to abduction at 90 degrees while the patient was in the supine position. The ultrasound probe was placed transversely just below the middle of the clavicle and the probe was tilted to the cephalad to scan the costoclavicular area. All 3 cords in a location lateral to the axillary artery were optimized in the ultrasound image (Figure 1b). The in-plane technique was used to check the presence of blood in the negative aspiration by passing through the gap between the lateral and posterior cords in the brachial plexus cluster. The needle was directed from lateral to medial. Approximately 1–2 mL of saline 0.9% was injected to confirm the position of the needle. Subsequently, 25 mL of LA containing a mixture of 1% lidocaine and 0.25% bupivacaine was injected. 

Performance time was defined as the time elapsed from the local skin infiltration to the end of LA injection. After the local anaesthetic injection, sensory and motor blocks were examined in every 5 min and recorded by a blind investigator for the onset time. Sensory block was defined as the sensation loss to cold (ice) in the cutaneous distribution of the median (MN), radial (RN), ulnar (UN) and musculocutaneous (MCN) nerves (cold examination; 2 = cold, 0 = no sensation) Motor blockade of each of the 4 nerves in the ipsilateral upper extremity was also rated and graded according to a 3-point qualitative scale (2 = normal motor power, 1 = paresis, and 0 = paralysis). Motor blockage of MN, UN, MCN, and RN was tested by thumb opposition with the index finger, thumb opposition with the little finger, elbow flexion and wrist extension, respectively. The block onset time, namely the primary endpoint was defined as the time needed to achieve ≥ 14 points after the end of LA injection through the block needle [3].

**Figure 2:**
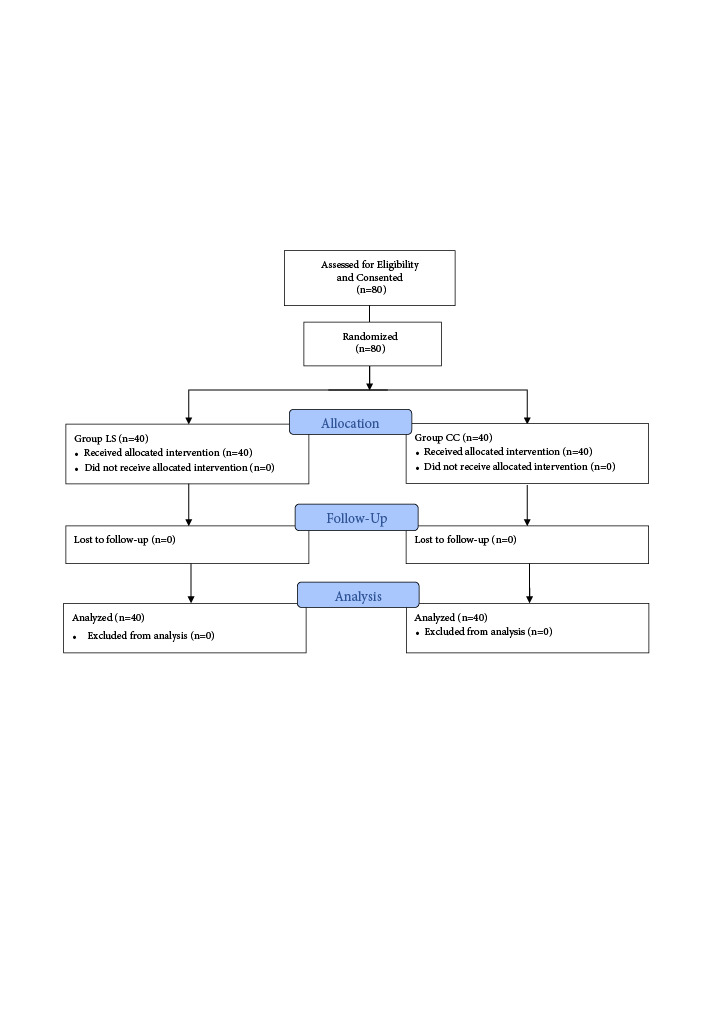
CONSORT flow diagram of the study. Group LS: Infraclavicular; Group CC: Costoclavicular.

The same blind observer decided which patients who were admitted to the operation room needed general anesthesia or rescue block or without any iv. narcotics for the surgical procedure. No sedation was used for the patients during the intraoperative period. The patients were followed up for 24 h postoperatively (Figure 2).

### 2.3. Statistical analysis

G*Power (version 3.0.10)16 was used to estimate the sample size (a priori). The overall “onset time the sensorimotor blockade” was used as the primary outcome variable. After a pilot study, it was estimated that a sample size of 30 patients would provide 80% power with an α error of 0.05. We recruited 40 patients per study group.

All statistical analyses were performed using the IBM SPSS for Windows version 20.0 (IBM Corp., Armonk, NY, USA) software. Kolmogorov–Smirnov test was used to assess the assumption of normality. Continuous variables were presented depending on normal distribution with either mean ± standard deviation or (in case of no normal distribution) median (25th–75th percentile). Categorical variables were summarized as counts (percentages). Comparisons of continuous variables between groups were carried out using either independent samples t-test or Mann–Whitney U test. Association between two categorical variables was examined by Chi-square test. A value of p < 0.05 was considered to be statistically significant.

## 3. Results

The demographic data and surgical parameters of both groups are presented in Table 1. No statistically significant difference was found between the two groups. Sensorimotor onset time was found to be faster in the CC group [(15.95 ± 2.97) min] compared to the LS group [(17.72 ±4.15) min]. There was a statistically significant difference between two groups in terms of sensorimotor onset time (p = 0.031, Table 2). There was no difference between two groups in terms of the block performance times and post-block motor block dissolution times. 

**Table 1:** Demographic data.

	Group- LS	Group- CC	p
Age (year)	33.98 ± 11.74	35.55 ± 17.36	0.636a
Sex (male/female)	18 / 22	21 / 19	0.502a
Weight (kg)	72.02 ± 14.69	70.95 ± 13.47	0.734a
BMI, kg/m2	24.35 (21.85 – 31.20)	23,50 (25.85 – 30.35)	0.513b
ASA (I/II/III)	32 / 6 / 2	31 / 7 / 2	0.955c
Types of surgery (hand/wrist/forearm/elbow)	17 / 9 / 8 / 6	15 / 10 / 11 / 4	0.789c
Duration of surgery (min)	59.50 ± 13.19	65.25 ± 22.86	0.172a

Data are presented as median (25%–75%), mean ± sd and patient numbers.a: T-test ; b: Mann–Whitney U test ; c: Chi-square test, ASA: American Society of Anesthesiologists Score, BMI: body mass index, min: minute.

**Table 2:** Clinical parameters.

	Group-LSn = 40	Group-CCn = 40	p
Imaging time (s)	8.00 (6.00–10.00)	7.00 (5.50–8.00)	0.207a
Performance time (s)	96.72 ± 30.23	90.02 ± 20.63	0.251b
Onset time (min)	17.72 ± 4.15	15.95 ± 2.97	0.031b *
Motor block durations (h)	4.50 (4.00–6.00)	5.00 (4.00–6.50)	0.137a

Data are presented as median (25-75 percentile) and mean ± sd, * p < 0.05, a : Mann–

Optimization time of the ultrasound image and block performance time was found to be faster in the CC group among the patients with BMI ≥ 30 in the study; however, no statistically significant difference was found between the two groups (Table 3).

**Table 3:** Clinical parameters of patients with body mass index ≥ 30.

	LS Approachn = 13	CC Approachn = 10	p
BMI ≥ 30 Imaging time (s)	8.38 ± 3.01	7.80 ± 3.58	0.675a
BMI ≥ 30 Performance time (s)	94.69 ± 28.79	89.60 ± 22.56	0.650a

Data are presented as mean ± sda: T-test , BMI: body mass index , s: second.

Brachial plexus block was successfully performed in all 80 patients included in the study, and surgical anesthesia was provided without any need for rescue block or additional opioid administration. None of the patients had a major complication and any neurological damage during the 24-h follow-up period.

## 4. Discussion

Our study findings have shown that ultrasound guided the CC approach provided a faster sensorimotor blockade compared to the LS approach. To the best of our knowledge, this study is the first one demonstrating that the CC approach provides a faster onset of a sensorimotor blockade compared to the LS approach when a mixed LA fluid of 1% lidocaine and 0.25% bupivacaine is used.

The costoclavicular block was described in 2015. In this approach, lateral, medial and posterior cords of the brachial plexus are tightly bundled together [3]. The ultrasound image was optimized to clearly view all three cords of brachial plexus lying lateral to axillary artery, and the needle was inserted with plane technique from the lateral to medial direction. In 2017, Nieuwveld et al. [7] defined the medial approach for the CC block. While defining the medial approach, it was stated that the coracoid process might be an obstacle in the lateral approach and direct the needle towards the pleura. In the CC approach, there are differences in the local anesthetic volume and dose as well as the differences in the direction of needle.

Leurcharusmee et al. [8] had stated that there was no difference between sensorimotor blockade times between CC and LS, whereas Karmakar et al. [9] reported that CC block provided faster sensory blockade. However, methods of the studies differ from each other at some points. The most important difference is the LA content and dose of the fluid used for the blockade in the procedure. Leurcharusmee et al. [8] used 35 mL of 1% lidocaine and 0.25% bupivacaine as the local anesthetic, whereas Karmakar et al. [9] used 25 mL of 0.5% ropivacaine in their study. Karmakar et al. [9] reported in their study that the use of high doses and mixed local anesthetic substances may affect the advantage of CC approach in faster block formation and that the results may differ accordingly. In our study, we observed that CC block and sensorimotor block were performed in a faster fashion by using a mixed local anesthetic substance containing 25 mL of 1% lidocaine and 0.25% bupivacaine. We believe that the dose of local anesthetic substance administered at this point is important, whereas adding a fast-acting local anesthetic substance such as lidocaine into the block fluid does not affect the potential advantages of CC block. 

In the minimum effective volume study conducted using the CC block, it was shown that 1.5% lidocaine with epinephrine 5 μg/mL and 34 mL for the minimum effective volume in 90 % of patients (MEV90) was found to be adequate [10]. While defining the block, it was stated that the block was active and effective with 20 mL of 0.5% ropivacaine, while CC was reported to be efficient in a small volume (20 mL) in a sonoanatomy study of block dynamics [3,11]. Ming et al. [12] reported that the MEV 90 of 0.5% ropivacaine required to produce surgical anaesthesia with an ultrasound-guided CC-BPB is 20.9 mL. Kewlani et al. [13] reported that the median effective dose for surgical anesthesia in 50 % of the patients (ED 50) is 13.5 mL and ED 95 is 18.9 mL of 0.5 % ropivacaine. Both Ming et al. [12] and Kewlani et al. [13], the standard block technique was used for the CC approach in their study. Variable types and volumes of local anesthetics, ranging from 20 to 40 mL, have been used for administering a successful infraclavicular brachial plexus block without creating a significant effect on surgical anesthesia [9–11]. However, randomized controlled local anesthesia volume, dose-comparative studies are needed for the CC approach. 

In our study, sufficient sensorimotor block was provided for surgical anesthesia after the block in both groups. However, there is not a standard definition of “onset time“or “being ready for the operation” after a peripheral nerve block. We also used the criteriafrequently used in regional anesthesia studies as in the studies of Karmakar et al. [9] and Leurcharusmee et al. [8] ; however, there may be differences in terms of methods used in the studies [11,14,15]. Despite these limitations and differences, we suggest that a faster sensorimotor block in the CC block group is an advantage in shortening the waiting period for the operation. In recent years, Leyare et al. [16] compared single injection and double injection technique in CC block and stated that there was shorter onset time and total anesthesia-related time in double injection technique. Considering this, further studies are needed with the injection technique related to CC block.

In the current study, performance time and US optimization times were similar in both groups. However, during this study, we realized that image optimization was achieved for obese patients in a shorter period of time using the CC block; therefore, we compared the performance and US optimization times between CC and LS groups in patients with a BMI ≥ 30. Although there was not any statistically significant difference, durations were shorter in the CC group. The advantages of the CC are that the cords are more superficial and related, and they can be seen in a single US window in the CC block approach. However, an important limitation of our study is that the number of patients with BMI ≥ 30 was limited in the study. Although the case report stating that the costoclavicular approach is advantageous and safe in obese patients in the literature supports this conclusion, randomized controlled studies are needed on this subject [17].

No significant complications was observed directly related to the technique or local anesthetic injection in the two study groups.

The main limitation of our study is that all patients monitored only for the first 24 h postoperatively; follow-up might be inadequate to determine neurological dysfunction. The body mass index of patients studied was low. Therefore, the results of our study may not apply to the obese, and future research should compare block dynamics between the CC and LS approaches in the obese. In addition, in this study, 25 mL of LA containing a mixture of 1% lidocaine and 0.25% bupivacaine was used for the LS approach, and the CC approach may be considered suboptimal, but there is no MEV90 data for bupivacaine and MEV90 for 1.5 % lidocaine is 34 mL. We chose to use 25 mL because this is the volume we typically use for the LS approach at our institution.

## 5. Conclusion

Ultrasound guided LS approach is a frequently used regional technique that has been shown to be effective and safe. However, we have demonstrated that the CC approach provides faster onset of sensory blockade and earlier readiness for surgery than the LS approach. It is important to evaluate the effectiveness and safety of the CC block in adults, pediatric and obese patient groups using randomized controlled trials.

## Informed consent

Informed consent was obtained from all individual participants included in the study.

All procedures involving human participants were performed in accordance with the ethical standards of the institutional and / or national research committee and with the 1964 Helsinki Declaration and its later amendments or comparable ethical standards.
